# New Appliances & Things Medical

**Published:** 1910-02-26

**Authors:** 


### NEW APPLIANCES & THINGS MEDICAL.

[We shall be glad to receive at our Office, 28 & 29 Southampton Street, Strand, London, W.C., from the manufacturers, specimens of all new preparations and appliances.]

### TUBERAL.

(Sole Agents for Great Britain: Paul Jahn and Co., 67-71 Pentonville Road, N.)

We have received literature and a sample bottle of "Tuberal," a substance the preparation of which is described only in very general terms, but which seems to be intended for the production of passive immunity in tuberculosis. It is stated to emanate from a laboratory near Berlin, presided over by a Dr. Thamm; but in the fullest German work on the treatment of consumption which we know of (Schröder's and Blumenfeld's "Therapie der Chronischen Lungenschwindsucht") we find no mention of Tuberal, although the book was published at the date of some of the numerous testimonials furnished, and the chapter on specific therapy in it was written by the medical director of a large sanatorium near Berlin. However, any who are inclined to a cautious trial in advanced cases can obtain a week's supply of Tuberal, at the cost of half-a-crown, on application to the sole agent—P. Jahn, 67 Pentonville Road, London.

### "LEUKOPLAST" ADHESIVE PLASTER.

(P. Beiersdorf and Co., Chemical Works, Hamburg. London Office: 7-8 Idol Lane, E.C.)

In Continental institutions, both public and private, this white rubber adhesive plaster is very well known; it is, however, not quite so familiar to English practitioners. Those who do not yet know its usefulness would do well to order a sample packet and try it for themselves. We have known the plaster and used it for some years, and find it a thoroughly trustworthy dressing in all cases where a strong, firm, non-irritating and durable outer covering is required. It is quite different from the ordinary adhesive plaster—that sticky, filthy composition spread on crinoline, which needs to be heated before it can be used. "Leukoplast" is always ready, always easy to apply, and always sticks. It is, therefore, a "pleasant plaster to use," sticking smoothly and easily, and needing little beyond ordinary attention to be applied successfully. Nor have we found that there are any material objections to its use. The delicate skins of children are often severely irritated by common adhesive plasters; but, in our opinion, there are few better dressings after an inguinal hernia operation in a child than a layer of plain xeroform gauze kept in place by a strip of perforated "Leukoplast." This is an economy of dressing and is a cleanly, practical way of dealing with a wound that with ordinary dressings needs a great deal of care to prevent it from becoming contaminated. "Leukoplast" is easy to remove, and it causes the patient no pain if the plaster is sharply ripped off. It is perhaps the finest adhesive for surgical work with which we are acquainted, and no general practitioner who has once used it is likely to undervalue its excellence. Messrs. Beiersdorf and Co. now possess a London office, from which samples and price-lists will be sent on application.

